# The impact of resilience on the mental health of military personnel during the COVID-19 pandemic: coping styles and regulatory focus

**DOI:** 10.3389/fpubh.2023.1240047

**Published:** 2023-08-09

**Authors:** Fei Cao, Juan Li, Wei Xin, Zhibing Yang, Di Wu

**Affiliations:** ^1^Department of Sociology, School of Law, Jiangnan University, Wuxi, China; ^2^Department of Medical Psychology, The Sixth Medical Center of PLA General Hospital, Beijing, China; ^3^Department of Military and Political Training, Army Academy of Armed Forces, Beng Bu, China; ^4^Department of Medical Psychology, Air Force Medical University, Xi’an, China

**Keywords:** military personnel, mental health, resilience, coping style, regulatory focus, COVID-19

## Abstract

Military personnel encountered multiple stressful events during the COVID-19 lockdown. Reducing non-combat attrition due to mental disorders is crucial for military morale and combat effectiveness. Grounded in stress theory and regulatory focus theory, this study investigates the influence of resilience on military personnel’s mental health; coping style and regulatory focus are considered potential mediators and moderators, respectively. We conducted a routine psychological assessment on 1,110 military personnel in China. The results indicate that: (1) resilience has a negative impact on the psychological symptoms of military groups; (2) mature and mixed coping styles in military personnel mediate the association between resilience and psychological symptoms; and (3) regulatory focus predominance has a negative moderating effect on mature coping styles’ effects on psychological symptoms. Furthermore, this study supports previous findings that resilience and mental health are interrelated; it demonstrates that military personnel can effectively reduce negative psychological symptoms by improving their resilience level and adopting mature coping styles under stressful situations. The current study presents interventional insights regarding coping styles and mental health from a self-regulatory perspective during the COVID-19 pandemic.

## 1. Introduction

The COVID-19 pandemic has brought about significant economic and health-related challenges, not just in terms of physical health, but also mental health and well-being (1). Several cross-sectional studies have revealed a correlation between the COVID-19 pandemic and higher-than-expected levels of mental distress in some populations, with depression, anxiety, and PTSD being the most frequently reported conditions (2).

During the COVID-19 pandemic, individuals in the military faced more sources of pressure than civilians. In addition to undertaking intensive training, living by strict military standards, and having little free time (3), military personnel may have also faced pressure related to family members falling ill, declining income, and social distancing requirements (4, 5). Due to their occupational characteristics, such as combat exposure and deployment tasks, military personnel already face a relatively high risk of developing mental illness (6). Thus, to reduce non-combat attrition, it is necessary to consider measures to prevent psychological symptoms from becoming psychological disorders (2). In light of the COVID-19 pandemic, scholars are increasingly focusing on the prevention of mental health issues, shifting their focus to the cultivation of psychological resources such as resilience and social support (4, 7). Prior research has shown that resilience and individual coping styles can effectively alleviate adverse psychological symptoms caused by stressors.

We aimed to shed light on the influence of resilience on the mental health of military personnel, taking coping style and regulatory focus as the intermediary and moderating variables, respectively. We further aimed to explore the mechanism by which military personnel reinforce their internal stress resources, providing evidence and support for psychological health interventions during the pandemic.

### 1.1. The effect of resilience on mental health

The concept of resilience was developed from research on crisis response and stress coping (8). It refers to an individual’s internal resources that enable their successful adaptation when facing adversity, trauma, threats, or other major life pressures (9). Military personnel often face higher mental health risks than do civilians owing to their occupational characteristics of chronic exposure to high-pressure environments (6). In the existing literature, topics related to the mental health of military personnel are often based on clinical outcomes, such as a high incidence of psychiatric problems (e.g., anxiety, depression, and posttraumatic stress disorder) and increased rates of suicide (10–12). Due to the significant increase in military personnel experiencing mental health problems, it is critical to develop strategies to prevent psychological symptoms from developing into more serious psychiatric problems (13).

Extensive research has confirmed that resilience reduces the likelihood of mental health issues (12, 14–17). Psychological resilience is seen as a positive psychological quality that can counteract the adverse effects of stressors, allowing individuals to experience fewer negative emotions, cope better in the face of unexpected events, and have a greater sense of subjective well-being (9, 18). In a study of frontline healthcare workers during the COVID-19 pandemic, resilience was found to be protective against psychological problems such as anxiety, depression, and burnout (19–21). In studies of military populations, resilience has also been found to promote better adjustment to deployment, as well as to reduce the risk of depression, anxiety, substance abuse, and suicide among soldiers (14, 22). Resiliency training improves soldiers’ rational understanding and ability to use more aggressive coping strategies in the face of stressors (23, 24). In studies of veterans, PTSD severity was lower in individuals with high (versus low) resilience; moreover, resilience factors influenced adaptive and coping behaviors and moderated the relationship between adverse experiences and psychiatric disorders (25–27). Thus, there is good reason to believe that resilience positively predicts mental health. In light of this, we formulated the following hypothesis:

*Hypothesis 1*: Psychological resilience positively predicts mental health in military personnel.

### 1.2. The mediating role of coping styles

Individuals facing stressful situations tend to employ different cognitive and behavioral skills to manage potential threats and effectively reduce the impact of stress and its accompanying adverse consequences for personal resources (28, 29). A growing number of scholars view mental health phenomena as processes by which resilience comes into play, with the outcomes determined by the interaction between personality traits and coping styles (30–32). Many studies have focused on the positive effects of resilience and coping styles on mental well-being outcomes (24, 33, 34). During the COVID-19 pandemic, scholars found that positive coping was associated with fewer stress symptoms, as well as decreased levels of anxiety, depression, PTSD, and other psychological disorders in healthcare workers, while negative coping was associated with more emotional stress and psychological symptoms (18, 21, 24). Appropriate coping style has a positive relationship with physical and mental health, quality of life, and subjective well-being (29, 35–37), suggesting that coping style may be an essential component in the mechanism by which resilience plays a protective role in mental health outcomes. Past studies have examined coping strategies as mediating factors in the relationship between resilience and adaptive outcomes such as somatic health symptoms and environmental adjustment (9). In studies on student populations, coping responses have been found to influence the impact of resilience on various outcomes, particularly physical health and college adjustment (9). Therefore, we posited the following:

*Hypothesis 2*: The impact of resilience on mental health is mediated by coping styles.

### 1.3. The moderating role of regulatory focus

Mental health outcomes under stress or adversity are often due to the interaction of factors in an individual’s complex ecosystem (38, 39). Regulatory focus refers to the specific tendencies that individuals exhibit in the process of self-regulation to achieve their desired end states (40). In response to specific situations, individuals adjust their cognition and behavior through a specific regulatory focus (40). Stress theory suggests that different stressors lead to different coping styles, implying that differences in personality and self-regulation may affect the strategies people use to reduce the discomfort caused by pressures (28, 41). The regulation focus theory suggests that individuals with a promotion-focused predominance are driven to pursue success and profit, pay more attention to positive information and results, view stressful situations as opportunities and challenges, and mobilize all resources available to achieve successful outcomes in their behavioral strategies. By contrast, prevention-focused individuals are risk-averse, sensitive to negative information and outcomes, seek safety and non-failure in their behavioral strategies, perceive stressful situations as threats and obstacles, and consume more of their internal resources in such situations (38–40, 42).

Current empirical studies indicate that different focal conditioning affects individuals’ choice of coping style, leading to different psychological experiences and behavioral outcomes. A promotion focus tends to be associated with positive and well-adapted coping styles, as well as positive emotional experiences with fewer psychological symptoms (29, 38). On the other hand, a prevention focus tends to be associated with passive coping styles, negative emotional experiences, and more maladaptive outcomes (43, 44). Thus, we developed the following hypothesis:

*Hypothesis 3*: The indirect effect of the degree of resilience on mental health through coping styles is moderated by regulatory focus.

**Figure 1:**
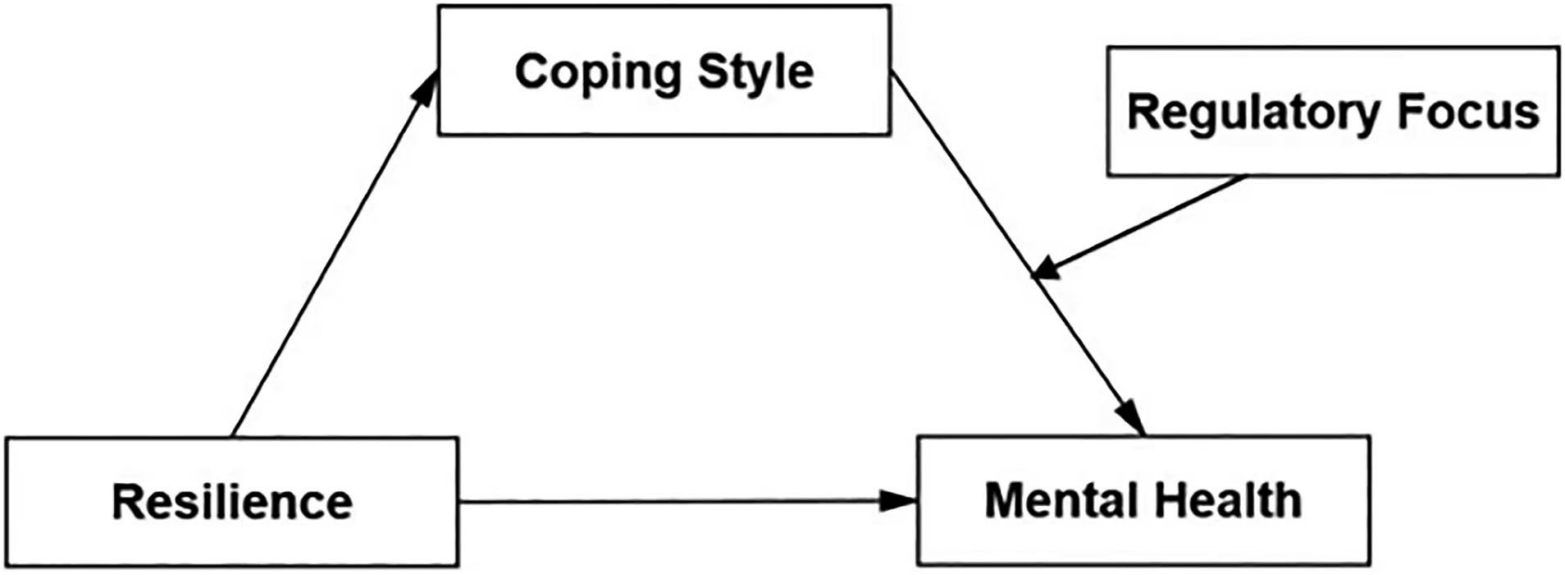
Theoretical framework.

The conceptual model utilized in this study is set out in Figure 1.

## 2. Methods

### 2.1. Participants and recruitment

The participants were military officers from grassroots units who took part in a routine psychological assessment to ensure that their psychological status at the time was suitable for the military environment and their job requirements (*N* = 1,206). These military officers were all on active duty and were stationed in the field. Their main task was to adapt to the field environment and daily military training to improve their field combat effectiveness. All participants were made aware of and consented to the study’s objectives, and 1,110 valid questionnaires were collected. The final sample included 1,110 participants with a 92.04% response rate.

### 2.2. Measures

#### 2.2.1. Demographics

We used a demographic information questionnaire to collect demographic data, including the following five parameters: (a) gender, (b) age, (c) personnel category, (d) education level, and (e) place of upbringing.

#### 2.2.2. Symptom checklist-90

We used a 90-item checklist, the symptom checklist-90 (SCL-90), to assess the participants’ mental health based on their self-report Likert scale responses (45). Higher scores suggest more severe psychological symptoms and therefore represent poorer mental health. The SCL-90 aims to assess the severity of individuals’ self-perceived symptoms across nine dimensions (i.e., somatization, obsessive-compulsive, interpersonal sensitivity, depression, anxiety, hostility, phobic anxiety, paranoid ideation, and psychoticism). Participants are asked to respond from 1 (*none at all*) to 5 (*very severe*) in terms of their experience of the symptom described by each item. The total score ranges from 90–450, with a higher score denoting more severe symptoms. The nine dimension scores provide insight into the characteristics of the symptom distributions and are a valuable tool for assessing mental health. The Cronbach’s *α* coefficients for the nine subscales in the current study ranged from 0.811 to 0.904.

#### 2.2.3. The Conner–Davidson resilience scale

We used the Conner–Davidson resilience scale (CD-RISC) to assess participants’ psychological resilience (46). The scale has 25 items, each of which is assessed on a 5-point Likert scale ranging from 0 (*completely false*) to 4 (*almost always true*) (1, *rarely true*; 2, *occasionally true*; 3, *often true*). Total scores range from 0 to 100 points, and higher scores indicate better resilience. In the current research, the Cronbach’s *α* coefficient of this scale was 0.975.

#### 2.2.4. Coping style questionnaire

We employed the coping style questionnaire (CSQ) to evaluate the kinds of coping strategies military personnel used during the pandemic (47). This questionnaire was developed according to Folkman and Bond’s coping and defense questionnaires (48, 49) and has primarily been used to assess coping styles in the context of Chinese linguistic features. The 62-item questionnaire consists of 6 subscales (problem-solving, self-blaming, help-seeking, fantasizing, escaping, and justifying), and each item is scored as either 0 (*agree*) or 1 (*disagree*). Problem-solving and help-seeking are recognized as mature coping styles; self-blaming, fantasizing and escaping are recognized as immature coping styles; and justifying is recognized as a mixed coping style. The Cronbach’s *α* coefficients for the six subscales in the current study ranged from 0.776 to 0.899.

#### 2.2.5. Regulatory focus questionnaire

We used the 11-item regulatory focus questionnaire to measure participants’ regulatory focus predominance (50). The questionnaire consists of a 6-item promotion focus subscale (e.g., “Do you often do well at different things that you try?”) and a 5-item prevention focus subscale (e.g., “Were you prone to getting on your parents’ nerves when you were a child?”) that we reverse scored. Participants rate each item from 1 (*rarely*) to 5 (*always*). In this study, the Cronbach’s *α* coefficients of the two subscales were 0.805 and 0.759. We calculated the predominant regulatory focus in the current study by subtracting the mean rating for prevention-related items from the mean rating for promotion-related items (51). Thus, we acquired an index of regulatory focus predominance, with a higher value indicating a tendency toward promotion predominance.

## 3. Data analysis and results

We utilized IBM SPSS 23.0 (IBM Corporation, Armonk, NY, United States) for statistical organization and analysis to investigate the connections between psychological symptoms, coping style, and resilience. We also conducted Pearson correlation analyses. We tested the mediating and moderating effects (models 4 and 14) through the SPSS macro program PROCESS 3.5, developed by Hayes (52, 53).

### 3.1. Descriptive statistics and correlation analysis

Table 1 presents sociodemographic descriptions. The participants were primarily male (94.68% of the total), with an average age of 25.12 ± 5.21 years old. In terms of education level, more than half had a high school degree or above (68.11%), and most grew up in rural areas (65.77%).

**Table 1:** Descriptive statistics of the participants (*N* = 1,110).

Variable	*N*	Percent (%)/mean ± SD
*Gender*		
Male	1,051	94.68%
Female	59	5.32%
*Personnel category*		
Commissioned officer	196	17.66%
NCO	544	49.01%
Compulsory serviceman	370	33.33%
*Age*		25.12 ± 5.21
*Education*		
High school	354	31.89%
Technical secondary school	437	39.37%
Bachelor’s degree	297	26.76%
Master’s degree or higher	22	1.98%
*Place of upbringing*		
City	380	34.23%
Countryside	730	65.77%

NCO, non-commissioned officer.

Table 2 displays the results of the correlation analysis. Resilience and psychological symptoms had a substantial negative association (*p* < 0.01), indicating that resilience was an important protective factor for mental health, and a high level of resilience can significantly reduce psychological symptoms. Mixed coping styles were positively correlated with the SCL-90 score (*p* < 0.01), and mature coping styles were negatively correlated with the SCL-90 score (*p* < 0.01). The results of correlation analysis between coping styles and psychological symptoms show that different coping styles have different effects on mental health. Compared with justifying, which represents the mixed coping style, individuals’ use of mature coping styles can significantly reduce their psychological symptoms. These findings support Hypothesis 1.

**Table 2:** Correlation of major factors and descriptive statistics.

Variable	*M* (SD)	1	2	3	4	5	6
1. SCL-90	106.49 (25.29)	—					
2. CD-RISC-25	65.55 (25.57)	−0.180^**^	—				
3. Mixed coping style	17.83 (3.00)	0.335^**^	−0.175^**^	—			
4. Mature coping style	10.73 (8.50)	−0.159^**^	0.369^**^	0.121^**^	—		
5. Immature coping style	4.68 (2.76)	0.004	0.007	0.044	−0.002	—	
6. Regulatory focus index	−0.36 (0.66)	−0.017	0.374^**^	−0.074^*^	0.310^**^	0.011	—

^*^p < 0.05 and ^**^p < 0.01.

### 3.2. Test for the mediating effect of coping styles

We conducted a bootstrap analysis with 5,000 resamples to evaluate the mediating effect of coping styles between resilience and psychological symptoms. Table 3 outlines detailed results. Resilience had a significant positive effect on mature coping styles (*β* = 0.369, *p* < 0.01) and a significant negative effect on mixed coping styles (*β* = −0.175, *p* < 0.01). Mature coping styles (*β* = −0.182, *p* < 0.01) and mixed coping styles (*β* = 0.349, *p* < 0.01) had a significant influence on psychological symptoms. Therefore, the relationship between resilience and psychological symptoms was mediated by coping styles (mature and mixed coping styles), indicating that coping styles were the mechanism by which resilience affected mental health. These results supported Hypothesis 2.

**Table 3:** Test for the mediating effect of coping styles.

Process	Variable	Model 4					
		*R*^2^	*F*	*β*	SE	*t*	95% CI
1. Mediator variable model (CS)							
MICS	Resilience	0.030	34.824^**^	−0.175	0.003	−5.901^**^	(−0.025, −0.013)
MCS		0.136	174.558^**^	0.369	0.003	13.212^**^	(0.037, 0.050)
IMCS		0.000	0.050	0.007	0.010	0.223	(−0.017, 0.022)
2. Dependent variable model (PS)	Resilience			−0.052	0.030	−1.701	(−0.111, 0.008)
	MICS			0.349	0.263	12.145^**^	(2.682, 3.715)
	MCS			−0.182	0.256	−5.991^**^	(−2.037, −1.032)
	IMCS			−0.012	0.082	−0.418	(−0.196, 0.127)
*R*^2^ = 0.155, *F* = 50.747^**^							

CS, coping style; MICS, mixed coping style; MCS, mature coping style; IMCS, immature coping style; PS, psychological symptoms. ^*^p < 0.05, ^**^p < 0.01.

Significant effects between the main variables were shown in bold.

### 3.3. Test for the moderating effect of regulatory focus predominance

We hypothesized that regulatory focus might moderate the indirect effect (the coping style-mental health pathway) of coping styles on mental health. The findings in Table 4 demonstrate that mature coping styles and regulatory focus were significantly associated with psychological symptoms (*B* = −0.894, *p* < 0.01); specifically, regulatory focus moderated the relationship between mature coping styles and mental health. The indirect effects of resilience on mental health through mature coping styles were moderated by regulatory focus. These results support Hypothesis 3.

**Table 4:** Results of the moderated mediation analysis.

Process	Variable	Model 14					
		*R*^2^	*F*	*B*	SE	*t*	95% CI
1. Mediator variable model (CS)							
MICS	Resilience	0.030	34.824^**^	−0.019	0.003	−5.901^**^	(−0.025, −0.013)
MCS		0.136	174.558^**^	0.043	0.003	13.212^**^	(0.037, 0.050)
IMCS		0.000	0.050	0.002	0.010	0.223	(−0.017, 0.022)
2. Dependent variable model (PS)	Resilience			−0.083	0.031	−2.635^**^	(−0.145, −0.021)
	MICS			3.135	0.268	11.689^**^	(2.609, 3.662)
	MCS			−1.967	0.283	−6.945^**^	(−2.523, −1.412)
	IMCS			−0.040	0.082	−0.486	(−0.200, 0.121)
	RFI			3.911	1.184	3.303^**^	(1.588, 6.234)
	CS × RFI						
	MICS×RFI			−0.015	0.390	−0.039	(−0.780, 0.750)
	MCS × RFI			−0.894	0.378	−2.364^*^	(−1.636, −0.152)
	IMCS×RFI			0.087	0.127	0.685	(−0.162, 0.336)
*R*^2^ = 0.169, *F* = 27.890^**^							

CS, coping style; MICS, mixed coping style; MCS, mature coping style; IMCS, immature coping style; PS, psychological symptoms; RFI, regulatory focus index. ^*^p < 0.05, ^**^p < 0.01.

Significant effects between the main variables were shown in bold.

**Figure 2:**
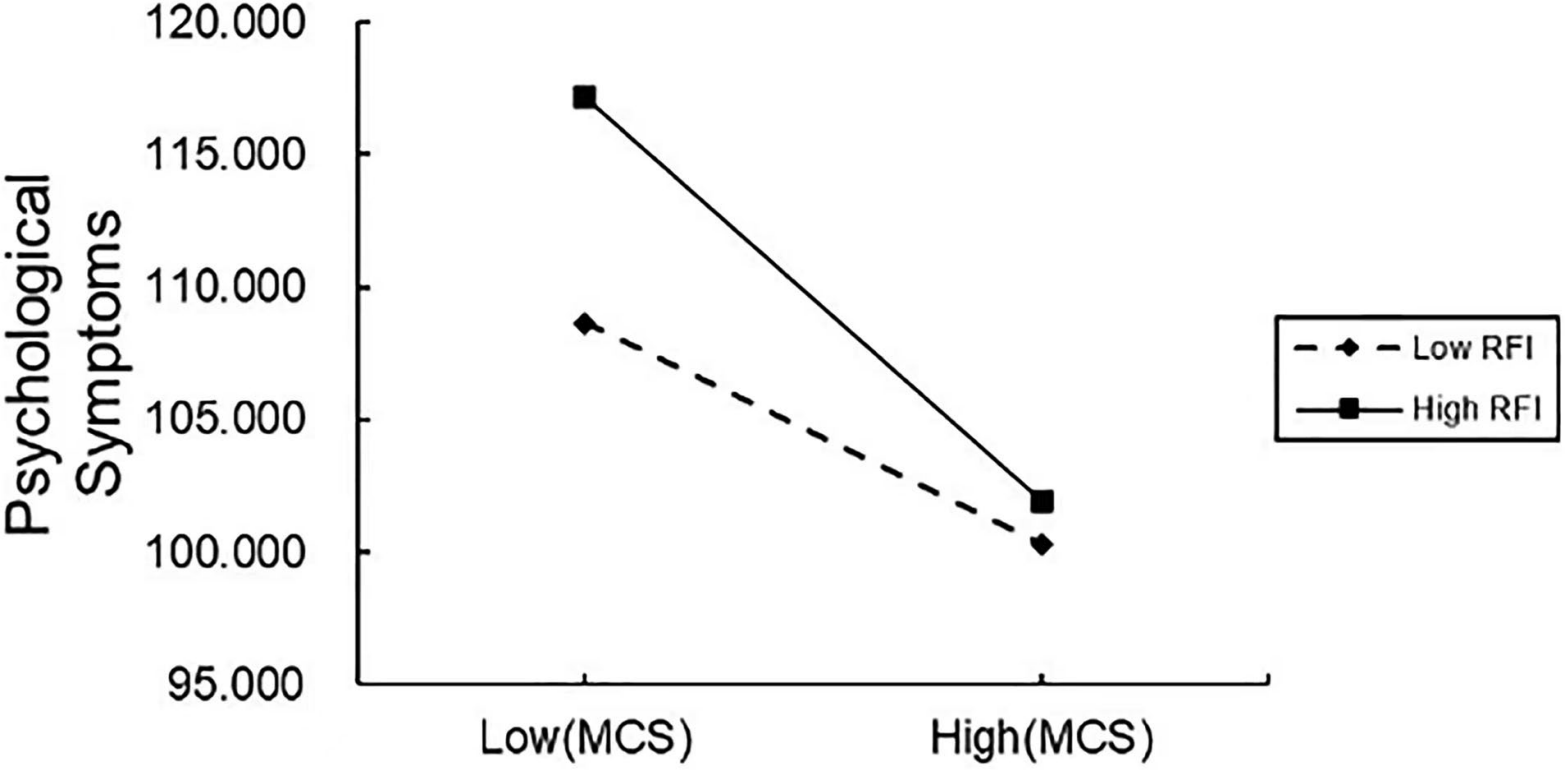
Moderated effect of regulatory focus on the relationship between psychological symptoms and a mature coping style. MCS, mature coping style; RFI, regulatory focus index.

To further interpret how coping style and regulatory focus interact, we performed a simple slope analysis (see Figure 2). For the military personnel with a high regulatory focus index, mature coping styles were negatively predictive of psychological symptoms (*B*_simple_ = −2.55, *t* = −5.88, *p* < 0.001). For those with a low regulatory focus index, the negative predictive effect of mature coping styles on psychological symptoms was diminished (*B*_simple_ = −1.39, *t* = −4.55, *p* < 0.001). This suggests that the tendency for mental health levels to improve with the use of mature coping styles rises significantly as the tendency to promote focus increases.

As shown in Table 5, resilience has a conditional indirect effect on psychological symptoms, mediated by mature coping styles at different values of the regulatory focus index. The indirect effect of mature coping styles was stronger at 1 standard deviation above the mean [*β* = −0.110, 95% CI (−0.169, −0.064)] than at 1 standard deviation below the mean [β = −0.060, 95% CI (−0.098, −0.029)].

**Table 5:** Results of the conditional indirect effect analysis.

Conditional indirect effect analysis	*β*	BootSE	BootLLCI	BootULCI
1 SD below the mean	−0.060	0.018	−0.098	−0.029
Mean	−0.085	0.020	−0.127	−0.052
1 SD above the mean	−0.110	0.027	−0.169	−0.064
Index of moderated mediation	−0.039	0.017	−0.075	−0.008

^*^p < 0.05, ^**^p < 0.01.

## 4. Discussion

Military personnel faced multidimensional stress during the COVID-19 pandemic, which increased their risk of developing psychological and psychiatric disorders (4). In the context of the pandemic, there was a decrease in the accessibility of timely and effective psychological interventions due to the prioritization of clinical treatments. In such instances of decreased resources, a shift in focus to protective factors for mental health is needed to prevent non-combat attrition and ensure combat readiness for the military population.

We investigated how resilience, coping styles, and regulatory focus influenced the mental health of military personnel during the COVID-19 pandemic. Resilience had a significant negative effect on psychological symptoms, and we identified the mediating roles of mixed and mature coping styles. Furthermore, regulatory focus negatively moderated the effect of mature coping styles on psychological symptoms.

### 4.1. Theoretical and practical implications

Our results have enriched the literature on the relationship between positive psychological resources and mental health outcomes in several ways. Firstly, the current study provides evidence and support for focusing on psychological services for military populations during the pandemic. Although previous studies have examined resilience and variables relating to mental health in military personnel, most of the literature is oriented toward the outcomes and occurrences of mental illness and focuses on clinical interventions (25, 27, 54, 55). In the context of public health emergencies, where medical resources are more centered on clinical treatments and psychological services are less accessible, the focus must be shifted to the prevention of mental illness and the mechanisms by which protective factors play a role. However, research on resilience in relation to mental health in pandemic contexts is still fairly limited for active-duty military personnel. The present study indicates that resilience has a positive effect on the psychological well-being of military personnel and serves as a protective factor for mental health in the pandemic context. This is consistent with past findings that resilience reduces negative outcomes from stressful events (19–21).

Secondly, we developed a conceptual framework in which we considered coping styles (mature, immature, and mixed) as mediating mechanisms that act on mental health through resilience. Specifically, we found that mature coping styles, such as problem-solving and help-seeking, were significantly and positively correlated with resilience and negatively correlated with psychological symptoms. We also found that mixed coping styles were significantly and negatively correlated with resilience but positively correlated with psychological symptoms. Immature coping styles, such as self-blaming and escaping, were not significantly correlated with resilience or psychological symptoms. Stress theory suggests that different stressors lead to different coping styles, and during the stress process, coping is highly correlated with emotion regulation. Specifically, certain coping strategies that avoid reality are always associated with adverse mental health outcomes, while other coping strategies have varying outcomes in different contexts (56), which is partially consistent with our findings. Notably, immature coping styles were not significantly correlated with either resilience or mental health outcomes in the current study. This may be related to the culture advocated by the military environment in which all military personnel are expected to function at a high level of proficiency in stressful situations (3). Negative or immature coping styles were the least commonly used coping strategies in relevant research with military personnel (3, 57). This suggests that encouraging the military population to adopt mature coping styles (i.e., help-seeking behaviors during stressful events) in military management and psychological services can better alleviate psychological symptoms in stressful situations.

Finally, we explored a critical boundary condition in the relationship between coping styles and psychological symptoms. The regulatory focus index was significantly and positively related to resilience, and mature coping styles were more effective at protecting mental health among military personnel with a high regulatory focus index. As the coping process is intricate and multifaceted, it is sensitive to environmental demands and resources as well as to personality traits that affect the perception of stress and use of resources for coping (56). In response to stressful situations, individuals adjust their cognition and behavior through two independent modes of self-regulation with distinct preferences for goal attainment and strategically different ways of coping: the promotion focus and prevention focus, respectively characterized by eagerness and by cautiousness and avoidance (40–42). Since a high regulatory focus index represents an individual’s preference for promotion-focused self-regulation, this implies that promotion-focused individuals can more successfully resist psychological threats arising from stressful events by enhancing their maturity-based coping skills. This finding can be explained by regulatory fit theory, which states that the effect occurring *via* the pursuit of goals matches self-regulation (58–60). Promotion-focused individuals are motivated by positive outcomes in the pursuit of goals and adopt more proactive strategies. This makes mature coping styles (such as problem-solving and help-seeking) match their goal-seeking strategy, resulting in better outcomes in stressful situations (27, 38) due to the regulatory fit effect.

### 4.2. Limitations and future research directions

Our research is restricted by some limitations. First, the study was cross-sectional, which means it can only reflect correlations among the variables. Future studies should examine causal patterns using longitudinal and experimental methods. Second, in previous studies on military populations, justifying was found to be significantly and negatively associated with help-seeking behaviors (31) and positively associated with negative coping styles (61). In the current study, however, justifying was positively associated with psychological symptoms and mature coping styles. This implies that justifying, as a mixed coping style, has a different working mechanism that significantly influences mental health outcomes when individuals cope with stressful events. Future studies should explore this association in greater depth. Finally, military personnel with different positions may have varying coping styles and levels of mental health. In the existing studies on Chinese military personnel, demographic variables, such as age, gender, education level, and military rank, are significantly correlated with mental health symptoms (e.g., anxiety, social anxiety disorder) (61–63). Moreover, in relevant studies conducted in western countries, sociodemographic characteristics, such as race, ethnicity, marital status, and enlistment age, can all affect the mental health of military personnel (64–66). As such, future research would benefit from focusing on specific personnel categories to develop more targeted guidance for psychological services.

## 5. Conclusion

We developed a moderated mediating model to explain the effects of resilience on the psychological well-being of military personnel. The current research has confirmed that coping styles—especially mature coping styles—play a fundamental role in the relationship between resilience and psychological symptoms in military populations, and may have been essential protective factors of mental health during the pandemic. Furthermore, this study indicates that promotion-focused individuals can more effectively resist the psychological threats associated with stressful events by enhancing the practice of mature coping styles. Besides advocating for a military culture which maintains the mental health of personnel, encouraging military members to contact significant others (e.g., telephone family and friends), as well as ask for advice or assistance from organization members when faced with specific problems, can benefit individuals’ successful adaption in stressful situations (3, 57). These findings offer insights and intervention strategies for mental healthcare in the military.

## Data availability statement

The datasets presented in this article are not readily available because in accordance with participant privacy and ethical requirements, we do not permit the sharing of data. Requests to access the datasets should be directed to caofei@jiangnan.edu.cn.

## Ethics statement

The studies involving humans were approved by Air Force Medical University evaluated and authorized the investigation, which was conducted with the informed consent of all participants. The studies were conducted in accordance with the local legislation and institutional requirements. Written informed consent for participation in this study was provided by the participants’ legal guardians/next of kin.

## Author contributions

FC performed the research and wrote the paper. WX and ZY assisted with the research and analyzed the data. JL provided technical guidance. DW conceptualized the current study and provided financial support. All authors contributed to the article and approved the submitted version.

## Funding

This study was supported by the General Project of Philosophy and Social Science Research of Colleges and Universities in Jiangsu Province (2021SJA0851) and the Fundamental Research Funds for the Central Universities (JUSRP12072).

## Conflict of interest

The authors declare that the research was conducted in the absence of any commercial or financial relationships that could be construed as a potential conflict of interest.

## Publisher’s note

All claims expressed in this article are solely those of the authors and do not necessarily represent those of their affiliated organizations, or those of the publisher, the editors and the reviewers. Any product that may be evaluated in this article, or claim that may be made by its manufacturer, is not guaranteed or endorsed by the publisher.
